# Evaluation of an AI-Assisted Colony Counting System Across Multiple Culture Media Using Standardized Pure Culture Plates

**DOI:** 10.3390/microorganisms14071426

**Published:** 2026-06-30

**Authors:** Xue Li, Meng Xiao, Dingding Li, Meihui Liu, Yingchun Xu

**Affiliations:** Department of Laboratory Medicine, State Key Laboratory of Complex Severe and Rare Diseases, Peking Union Medical College Hospital, Chinese Academy of Medical Sciences and Peking Union Medical College, Beijing 100730, China; lixue35@pumch.cn (X.L.); cjtcxiaomeng@aliyun.com (M.X.); 18401661736@163.com (D.L.); meihui0916@163.com (M.L.)

**Keywords:** colony counting, artificial intelligence, clinical microbiology, culture media, automated image analysis

## Abstract

Automated AI-assisted colony counting may improve standardization in digital microbiology, but performance can be affected by colony density, culture medium, colony morphology, adhesion, and plate artifacts. We evaluated the Starry-300 AI colony counting system using 382 standardized pure culture bacterial and yeast plates across four agar media. AI-assisted counts were compared with a three-reader median ImageJ-assisted manual comparator derived from independent counts by experienced technologists. The AI workflow showed close agreement with the manual consensus comparator across a broad colony density range. Overall, 360/382 plates (94.24%) were within ±10 CFU and 377/382 plates (98.69%) were within ±30 CFU of the manual median count. Error-based and agreement analyses showed a mean absolute error of 3.19 CFU/plate; both the intraclass correlation coefficient and Lin’s concordance correlation coefficient were0.99. AI software analysis required approximately 5–15 s/plate, although this did not include plate handling, correction, or reporting. These findings support the analytical feasibility of reviewable AI-assisted colony enumeration under controlled pure culture conditions. Further validation using primary clinical specimens, mixed cultures, near-threshold samples, and external sites is required before broad clinical implementation.

## 1. Introduction

Enumeration of microbial colonies as colony forming units (CFU) remains a foundational quantitative procedure in microbiology. Plate counts are used in clinical microbiology, antimicrobial susceptibility workflows, environmental monitoring, food testing, pharmaceutical quality control, and experimental microbiology [1,2]. In clinical microbiology, colony counts are not only analytical measurements but also part of specimen-specific interpretation. For urine cultures, laboratories commonly report growth by order of magnitude categories, such as 10^2^, 10^3^, 10^4^, and 10^5^ CFU/mL, and the clinical meaning of these categories depends on symptoms, specimen collection method, sex, risk group, and whether the culture is more consistent with infection, colonization, or contamination [3,4]. For bronchoalveolar lavage (BAL) and other quantitative lower respiratory specimens, CFU/mL thresholds are also used, with 10^4^ CFU/mL commonly used as a BAL reference threshold in many protocols [5,6]. Therefore, colony counting in clinical microbiology is both a measurement step and a judgment-dependent interpretive step.

Digital image analysis was introduced to reduce the subjectivity and workload of manual enumeration, and approaches have evolved from classical thresholding and edge detection to machine learning and deep learning methods [7]. Open-source systems such as OpenCFU and commercial automated counters have demonstrated that automated colony detection can improve throughput and standardization in many settings, although performance may decline without visual correction when colony density, contrast, or separation is unfavorable [8,9].

More recent algorithms have attempted to address these limitations using high-throughput edge detection pipelines, synthetic image generation, and convolutional neural networks [10,11,12]. These studies collectively show that AI can improve colony detection, but they also highlight the importance of representative datasets, reviewable outputs, and validation under real-world visual challenges. In particular, colony counting is not merely a binary detection problem: algorithms must resolve colonies that vary in size, texture, boundary contrast, color, hemolysis, confluence, and spatial distribution across different media.

In parallel, clinical microbiology laboratories have adopted total laboratory automation (TLA), digital plate imaging, and algorithm-assisted interpretation to improve standardization and turnaround time [13,14]. Integrated digital microbiology platforms, including WASPLab, APAS Independence, and WASPLab-associated PhenoMATRIX or PhenoMATRIX PLUS algorithms, have been evaluated for clinical culture plate interpretation, urine culture screening, significant growth categorization, surveillance culture screening, image-based segregation and technologist review prioritization [15,16,17,18,19,20,21]. More recent AI systems have further extended automated interpretation to broader diagnostic culture plate reading and bacterial growth monitoring in clinical bacteriology [22,23]. However, these platforms are primarily designed for culture plate interpretation and workflow decision support rather than stand-alone quantitative colony enumeration across multiple organism groups, agar media, and colony density ranges.

Commercially available automated colony counting systems such as Interscience have been used for quantitative CFU enumeration in medical microbiology research [24]. More recently, AI-assisted versions such as the Scan Ai series have been introduced for automated colony enumeration, although peer-reviewed clinical evaluations of these stand-alone AI counters remain limited. For these systems, the central evaluation question is not only whether colonies can be detected, but also whether quantitative counts remain accurate and reproducible under visually challenging conditions, including dense growth, adherent or merged colonies, low-contrast boundaries, and media-dependent visual effects.

Existing studies and reviews have described automated plate interpretation, clinical microbiology AI applications, and dataset-based colony counting approaches [25,26]. However, fewer studies have evaluated AI-assisted colony enumeration using an independent multi-reader manual comparator while simultaneously stratifying performance by colony density, culture medium, and organism group and reporting correlation, error-based metrics, agreement statistics, and residual visual failure modes. Such an evaluation framework is important because colony counting performance may be affected by colony density, culture medium, colony morphology, weak colony separation, low-contrast boundaries, and small or crowded colonies [7,9,26].

Against this background, the present study evaluated the Starry-300 AI colony counting system in a challenge-driven setting. The study was designed to test whether a single AI-assisted workflow could maintain accurate and reproducible colony enumeration across multiple clinically relevant bacterial and yeast groups, multiple agar types, broad colony density ranges, and representative visual challenges, while preserving reviewability through digital images and detection overlays.

## 2. Materials and Methods

### 2.1. Study Design and Evaluation Framework

This was a single-center, single-platform method comparison and workflow evaluation study of an AI-assisted colony counter conducted at Peking Union Medical College Hospital using one Starry-300 imaging system and a standardized image acquisition protocol. The evaluation used a predefined challenge-driven framework covering organism group, culture medium, colony density, colony morphology, and reader variability. The primary analytical comparison was between AI-assisted counts and a manual comparator derived from three independent ImageJ-assisted manual counts. For each plate, the median of the three independent manual counts was first used as the initial manual count. When the absolute difference between the AI-assisted count and the initial three-reader median manual count exceeded 10 CFU, the case was flagged for joint consensus review by the three technologists, and the adjudicated consensus count was used as the final manual comparator. For plates without >10 CFU AI–median discrepancy, the three-reader median was retained as the final consensus-based manual comparator. This approach was adopted to reduce individual observer bias associated with a single manual reader.

**Figure 1:**
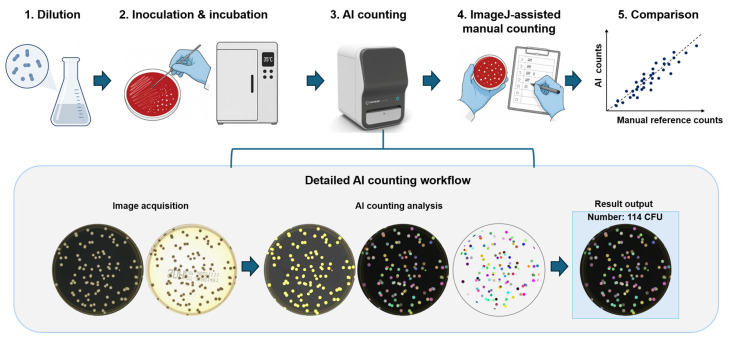
Study design, AI-assisted counting workflow, and dataset composition. The study workflow included strain selection and dilution, plate inoculation on different culture media, incubation, image acquisition, AI-assisted colony analysis, AI counting output, ImageJ-assisted manual counting by three technologists, and statistical comparison using the three-reader median manual comparator or consensus count (for plates with >10 CFU discrepancy between the AI and median manual counts). Plates were imaged under front- and back-light illumination, after which the AI system generated colony detection overlays and count outputs for visual review. Colored overlays represent AI-detected colony masks. In the multicolor instance maps, colors are arbitrary pseudo-colors assigned only to distinguish individual segmented colony instances, particularly in adherent or merged colonies. The gray alphanumeric markings visible in the plate image are manufacturer-printed product/lot information on the underside of the Petri dish. They represent non-biological background artifacts and were not counted as colonies, illustrating that the Starry-300 AI workflow could tolerate this type of plate-background marking in this example.

The complete workflow is summarized in Figure 1. Briefly, clinical isolates were selected, suspensions were prepared and serially diluted, plates were inoculated and incubated, images were acquired using the Starry-300 system, AI-assisted counting outputs were generated, manual reference counts were obtained from the same digital images, and statistical agreement analyses were performed.

### 2.2. Bacterial and Yeast Strains and Culture Conditions

Clinical isolates were obtained from the clinical microbiology laboratory of Peking Union Medical College Hospital. The isolate panel listed in the study protocol comprised 62 isolates: 10 *Escherichia coli*, 10 *Klebsiella pneumoniae*, 6 *Pseudomonas aeruginosa*, 6 *Acinetobacter baumannii*, 5 *Enterococcus faecalis*, 5 *Enterococcus faecium*, 5 *Staphylococcus aureus*, 5 *Staphylococcus epidermidis*, 5 *Streptococcus agalactiae*, and 5 *Candida albicans*. Strain selection was intended to include common clinical colony phenotypes, including mucoid colonies, small-colony appearances, hemolytic patterns, and colonies with different degrees of adhesion or confluence.

All strains were stored at −80 °C before testing. Before experiments, isolates were subcultured twice on Columbia blood agar plates (Oxoid, Thermo Fisher Scientific, Waltham, MA, USA) at 35 °C for 24 h to ensure purity and viability.

### 2.3. Preparation of Inoculum and Plate Inoculation

Fresh colonies were suspended in sterile saline to prepare microbial suspensions. Turbidity was adjusted and measured using a DensiCHEK Plus turbidimeter (bioMérieux, Marcy-l'Étoile, France). Bacterial suspensions were adjusted to 0.5–0.6 McFarland (McF), corresponding approximately to 1.5 × 10^8^ CFU/mL, whereas *C. albicans* suspensions were adjusted to 1.8–2.2 McF, corresponding approximately to 5 × 10^6^ CFU/mL.

Serial dilution was performed to generate a wide range of colony counts. Standardized suspensions were subjected to 1:10 serial dilutions in sterile LB broth. To refine the expected colony density, additional 1:1 and 1:2 dilutions of the 10^5^ CFU/mL suspension were prepared, producing final suspensions approximately in the range of 5 × 10^4^ to 10^3^ CFU/mL.

For each selected dilution, 10 µL was inoculated onto the appropriate agar plate using single-use calibrated 10 µL inoculation loops (Copan, Brescia, Italy). Replicate plates were inoculated at selected dilution levels to populate the target density gradient, including approximately 10, 50, 100, 300, and 500 CFU/plate. The suspension was spread evenly by rotating the plate approximately 60° three times. Plates were air-dried under a laminar-flow hood for 15 min and incubated at 35 °C for 18–24 h. After exclusion of non-informative or off-target plates during dataset assembly, 382 plates were included in the final analysis.

### 2.4. Selection of Culture Media

Culture media were selected to represent routine clinical and experimental agar backgrounds. Gram-negative bacilli were inoculated onto Columbia blood agar (BAP), MacConkey agar (MAC), and nutrient agar (NA). Gram-positive cocci were inoculated onto BAP and NA. *C. albicans* was inoculated onto Sabouraud dextrose agar (SDA). All culture media were sourced from Oxoid (Thermo Fisher Scientific, Waltham, MA, USA).

### 2.5. Automated AI Colony Counting

After incubation, all plates were imaged and analyzed using the Starry-300 automated colony counting system and its built-in AI analysis software (Jieyi Biotechnology Co., Ltd., Suzhou, China). Each plate was placed in the Starry-300 imaging chamber and images were acquired under the standard colony counting mode. The system used dual illumination, consisting of upper reflected light and lower transmitted light imaging, to capture complementary colony appearance information. The acquired plate images were exported in JPG format for documentation and manual review, whereas AI-assisted counts were generated by the built-in Starry-300 software.

The AI-assisted counting workflow was implemented as a multi-model deep learning pipeline within the Starry-300 software. In brief, the pipeline first localized the agar plate region of interest and then performed colony foreground segmentation, boundary refinement, small-colony segmentation, and colony-level instance segmentation. The segmentation components included U-Net- or U-Net++-based models for plate localization, whole-plate semantic segmentation, boundary refinement, and small-colony segmentation, together with YOLOv8n-seg models for instance segmentation of large- and small-colony regions. The semantic segmentation, boundary refinement, and instance segmentation outputs were integrated to generate the final colony instances and total colony count.

The software also included two YOLOv8s-cls classification models for workflow-related image category screening and countability assessment. These classification outputs were used for workflow routing, image state recognition, and threshold selection in the counting pipeline. They were not used for bacterial species identification, taxonomic classification or antimicrobial resistance prediction.

During model development, the Starry-300 AI workflow consisted of eight independently trained submodels: disk_segs, all_segs, edge, small_segs, large_insts, small_insts, stage1_5cls, and stage2_8cls. The five colony segmentation or instance segmentation models used dual-light inputs derived from upper and lower illumination images, whereas the upstream plate localization model and the two workflow classification models used upper-light images. Model-specific training and validation sample sizes were 1165/292 for disk_segs, 4655/1165 for all_segs, 37,940/8998 for small_segs, 12,001/3001 for edge, 10,438/2600 for large_insts, 24,430/6108 for small_insts, 13,144/3286 for stage1_5cls, and 13,270/3317 for stage2_8cls. These values represent model-specific annotated training samples, including ROI crops or image tiles, rather than independent whole-plate images. Detailed model architecture and training settings are provided in Supplementary Table S1.

Data augmentation was applied according to the model task. The U-Net/U-Net++ colony segmentation models used geometric and photometric augmentation, including flipping, rotation/shift/scale transformation, random cropping, and brightness/contrast adjustment. For dual-light segmentation models, upper- and lower-light images were augmented synchronously to preserve pixel-level alignment. The plate localization model used stronger augmentation to improve robustness to plate position variation, reflections, stains, and edge artifacts. The YOLOv8n-seg models used segmentation-oriented augmentation with mosaic disabled to avoid disrupting colony spatial distribution, whereas the YOLOv8s-cls models used conservative augmentation to preserve colony color, morphology, and texture.

Model development performance was evaluated on validation sets using task-appropriate metrics. For the U-Net/U-Net++ models, the best validation IoU and F1/Dice values were 0.99 and 0.99 for disk_segs, 0.89 and 0.94 for all_segs, 0.84 and 0.89 for small_segs, and 0.65 and 0.77 for edge. The lower IoU of the edge model was expected because its target consisted of narrow colony and plate-edge boundaries with a small pixel proportion. For the YOLOv8n-seg instance segmentation models, the mask mAP@50 and mask mAP@50–95 were 0.95 and 0.83 for large_insts, and 0.98 and 0.80 for small_insts. For the YOLOv8s-cls classification models, the validation top-1 accuracies were 1.00 for stage1_5cls and 0.97 for stage2_8cls.

The 382 plates included in the present evaluation dataset were used only for locked system-level performance assessment. They were not used for model retraining, fine-tuning, hyperparameter optimization, or model selection.

### 2.6. Manual Colony Counting and Time Measurement

Manual counting was performed by three independent experienced medical technologists. To ensure consistency of the visual input and to avoid differences caused by repeated physical handling, all manual counts were performed from the digital images acquired by the Starry-300 system. Counts were performed using the Cell Counter plugin in ImageJ software (National Institutes of Health, USA; version 1.53k). Each technologist manually clicked every visible colony in the image, and ImageJ accumulated the count.

The time required for each manual count was recorded using a stopwatch, beginning at the first click and ending when the technologist completed colony marking on the digital plate image. Thus, manual timing represented ImageJ-assisted colony clicking time only and did not include image opening, file handling, secondary review, result transcription, or reporting. This process was repeated for all 382 plate images by all three technologists, generating three independent manual counts and three independent manual time measurements per plate.

The three independent manual counts were also used to assess inter-reader variability, and plates with a maximal inter-technologist discrepancy greater than 30 CFU were identified as cases with high manual reader disagreement.

### 2.7. Statistical Analysis

Linear regression was used to describe the linear association between AI-assisted counts and the three-reader median manual comparator. Because correlation-based measures alone do not establish agreement, method agreement was further assessed using Bland–Altman analysis, absolute error metrics, Lin’s concordance correlation coefficient, the absolute agreement intraclass correlation coefficient (ICC), Passing–Bablok regression, and categorical agreement statistics. Ordinary least squares regression equations, R^2^ values, and 95% confidence intervals for slopes and intercepts were calculated using GraphPad Prism 10 (GraphPad Software, Boston, MA, USA). R^2^ was interpreted as a measure of linear association rather than as stand-alone evidence of agreement. Bland–Altman analysis was used to estimate mean bias and approximate limits of agreement (mean ± 1.96 SD). Passing–Bablok regression was additionally performed as a non-parametric method comparison analysis to evaluate constant and proportional bias between AI-assisted and manual counts.

Absolute AI–manual median count differences were summarized using predefined pragmatic image-level analytical thresholds. A difference within ±10 CFU was used as a stringent near-exact agreement threshold, whereas a difference greater than 30 CFU was used as a major analytical discrepancy threshold for error review. The >30 CFU threshold was also aligned with the inter-technologist variability analysis, in which a maximal difference greater than 30 CFU among manual readers was considered a large reader discrepancy. These thresholds were selected for analytical method comparison and failure mode screening and should not be interpreted as universal clinical decision thresholds. Differences among the three manual readers were assessed using the Friedman test for repeated non-parametric measures, with the exact *p*-value reported when available.

For each plate, the signed difference was defined as AI count minus the three-reader median manual count, and absolute error was defined as the absolute value of this difference. Mean absolute error (MAE), median absolute error (median AE), root mean square error (RMSE), and relative absolute error were calculated overall and by predefined colony density strata. Relative absolute error was calculated as |AI count—three-reader median manual count|/three-reader median manual count × 100%. Four-category agreement between AI and manual median counts was assessed using predefined categories of 0–50, 51–150, 151–300, and >300 CFU/plate. Exact category agreement, adjacent category shifts, and shifts by two or more categories were tabulated. Cohen’s kappa and quadratic weighted Cohen’s kappa were calculated to assess categorical agreement beyond chance. Confidence intervals for mean bias were calculated using the standard error of the mean, whereas confidence intervals for MAE, RMSE, ICC, Lin’s concordance correlation coefficient, Passing–Bablok slope estimates, and kappa statistics were estimated by bootstrap resampling. A post hoc failure mode review was performed for plates with an absolute AI–manual median discrepancy greater than 10 CFU. Each discrepant plate was reviewed using the original digital image and AI overlay, and one dominant visual factor was assigned from six predefined categories: irregular colony shape, merged/adherent colonies, uneven colony size, low boundary contrast, colony hemolysis or hemolytic halo, and excessive colony density.

## 3. Results

### 3.1. Dataset Composition and Overall Agreement

**Figure 2:**
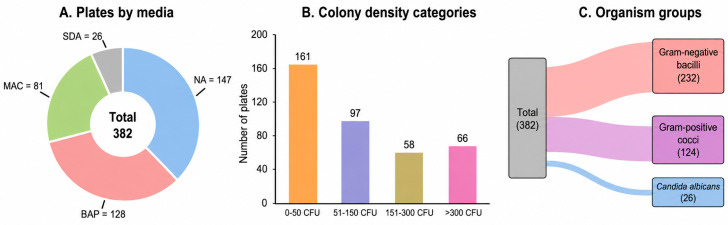
Dataset composition of the colony counting evaluation. The dataset included 382 plates distributed by culture medium, organism group, and colony density category. Culture media included BAP, MAC, NA, and SDA. Colony density categories included 0–50 CFU, 51–150 CFU, 151–300 CFU, and >300 CFU per plate.

A total of 382 agar plates were included in the full dataset for dataset composition and manual reader variability analyses. The dataset comprised 128 BAP plates, 81 MAC plates, 147 NA plates, and 26 SDA plates. Colony density categories were broadly distributed: 161 plates had 0–50 colonies, 97 plates had 51–150 colonies, 58 plates had 151–300 colonies, and 66 plates had >300 colonies, with the highest observed count reaching 1243 CFU/plate (Figure 2).

**Figure 3:**
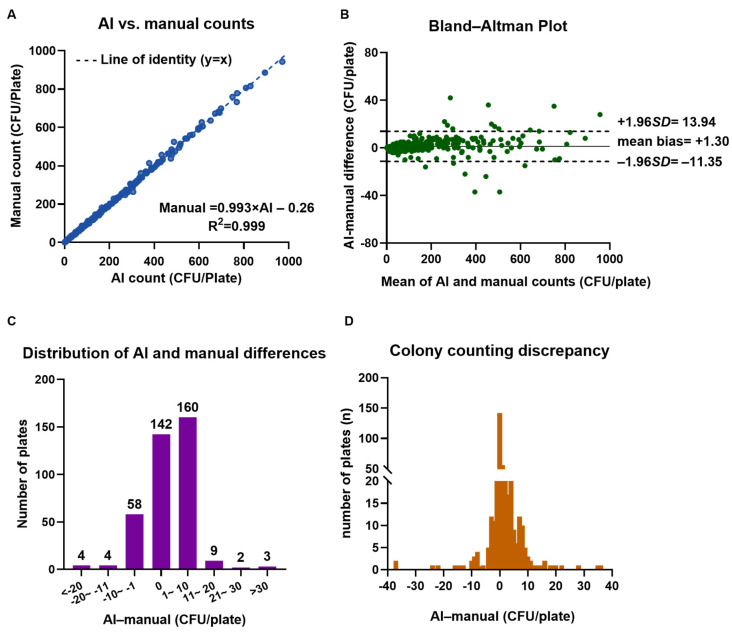
Agreement between AI-assisted counting and the three-reader median manual comparator. (**A**) Scatter plot showing the relationship between AI-assisted colony counts and three-reader median manual comparator counts in the 382 plates with paired AI and manual median data. The dashed line represents the line of identity, and the fitted regression line indicates the overall linear association between the two methods. (**B**) Bland–Altman analysis showing a mean AI–manual median bias of +1.30 CFU/plate and 95% limits of agreement from −11.35 to +13.94 CFU/plate. (**C**) Distribution of AI–manual median count differences by predefined discrepancy categories. (**D**) Histogram of AI–manual median count differences, showing that most paired plates clustered near zero difference and that large discrepancies were uncommon.

Overall agreement between AI-assisted counting and the three-reader median manual comparator count was assessed in the 382 plates with paired AI and manual median counts (Supplementary Table S2). The overall regression equation was Y = 0.99X − 0.26, with R^2^ = 1.00 (Table 1; Figure 3A). AI counts were identical to the consensus manual comparator for 142/382 paired plates (37.17%). A total of 360/382 paired plates (94.24%) differed from the manual median comparator by no more than 10 colonies. Only 5/382 paired plates (1.31%) had an absolute AI–manual median discrepancy exceeding 30 colonies (Figure 3C,D). Together, the scatter plot, discrepancy categories, and histogram in Figure 3 show that most paired results clustered near exact agreement, whereas large AI–manual median disagreements were uncommon.

Bland–Altman analysis of the 382 paired records showed a mean AI–manual median bias of +1.30 CFU/plate, with 95% limits of agreement from −11.35 to +13.94 CFU/plate (Figure 3B). Twenty paired plates (20/382, 5.24%) fell outside the approximate limits of agreement.

### 3.2. Performance Across Colony Density Ranges

AI-assisted counts maintained a strong linear relationship with the three-reader median or consensus (for >10 CFU discrepancy) manual counts across colony density categories in the paired AI–manual median dataset (Table 1). For paired plates with 0–150 CFU, the regression equation was Y = 0.98X + 0.31 (R^2^ = 1.00). For paired plates with 151–300 CFU, the regression equation was Y = 0.92X + 12.46 (R^2^ = 0.98). For paired plates with >300 CFU, including counts up to 1243 CFU/plate, the regression equation was Y = 0.99X + 3.75 (R^2^ = 0.99). Thus, agreement remained strong across density categories, although absolute error increased at higher colony burdens.

### 3.3. Accuracy for Different Microbial Groups and Species

Organism group regression analyses in Table 1 were performed on the paired AI–manual median records available for each organism group. AI-assisted counting showed high agreement with the three-reader median manual comparator across these organism groups. For Gram-negative bacilli, the regression equation was Y = 0.99X − 0.42 (R^2^ = 1.00). Individual Gram-negative species showed strong performance, although discrepancies were mainly associated with heavily adherent or overlapping colonies. For *P. aeruginosa* or *K. pneumoniae*, the system could identify two distinct colony size phenotypes on the same MAC plate and display the counts separately.

For Gram-positive cocci, the regression equation was Y = 0.99X + 0.61 (R^2^ = 1.00). *Staphylococcus* spp. showed the highest agreement, with slopes close to 0.99, followed by *Enterococcus* spp. at approximately 1.00. *Streptococcus agalactiae* showed a slightly lower slope (0.96) than the other Gram-positive cocci. This deviation was mainly associated with small-colony size and weak separation between excessively adjacent colonies. In these cases, neighboring colonies were sometimes grouped as a single instance, leading to mild undercounting. One *S. agalactiae* culture was among the plates outside the Bland–Altman limits of agreement.

For *C. albicans* on SDA, the regression equation was Y = 1.00X − 1.53 (R^2^ = 1.00), across a manual median count range of 7–1243 CFU. Among the nine SDA yeast plates with >300 colonies, all AI–manual median differences were within 30 CFU, although 3/9 fell outside the updated Bland–Altman limits of agreement. These findings indicate favorable performance in this limited *C. albicans* subset.

### 3.4. Performance Across Culture Media and Visual Morphologies

AI-assisted counting remained robust across the four culture media evaluated. The regression equations were Y = 0.99X + 0.28 (R^2^ = 1.00) for BAP, Y = 0.99X − 0.79 (R^2^ = 0.99) for MAC, Y = 0.99X + 0.15 (R^2^ = 1.00) for NA, and Y = 1.00X − 1.53 (R^2^ = 1.00) for SDA. Although slopes varied slightly among media, all media showed strong agreement with the three-reader median manual comparator. Common plate artifacts, including scratches and bubbles, were consistently ignored during AI analysis and did not visibly affect the count output.

### 3.5. Inter-Technologist Variability in Manual Counting

**Figure 4:**
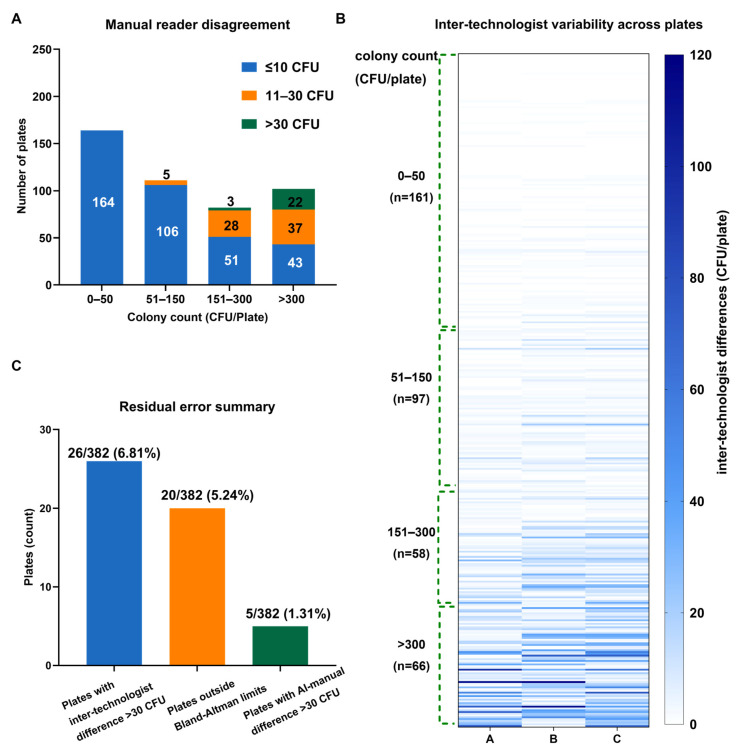
Manual reader variability and residual discrepancy patterns. (**A**) Stacked bar chart shows that inter-technologist disagreement increased with colony density. Low-density plates showed mostly small differences among technologists, whereas plates with 151–300 CFU and especially >300 CFU contained a higher proportion of moderate or large discrepancies. (**B**) Heatmap of inter-technologist variability across plates ordered by colony count category, illustrating that technologist disagreement concentrated mainly in high-density plates. In the heatmap, columns A, B, and C represent the three independent medical technologists who performed ImageJ-assisted manual colony counting. (**C**) Residual-error summary shows the number of plates with large inter-technologist discrepancy (26/382 plates with >30 CFU maximal inter-technologist difference), plates outside the Bland–Altman limits (20/382), and plates with large AI–manual median discrepancy (5/382).

Manual counting showed substantial variability among the three technologists. The maximal inter-reader discrepancy exceeded 30 CFU in 26/382 plates (6.81%), mainly among plates with high colony counts or visually complex growth (Figure 4A,C). Figure 4B shows that manual disagreement increased with density and was concentrated in visually complex plates. The Friedman test confirmed significant differences among the three manual readers (*p* = 0.0011). In contrast, repeated AI analysis of the same images produced identical results, indicating deterministic reproducibility.

### 3.6. Counting Time Efficiency

**Figure 5:**
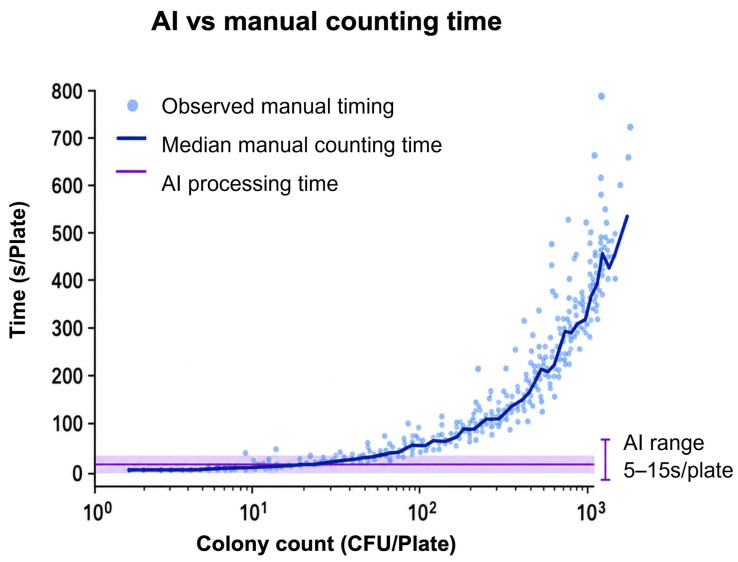
Software analysis time of AI-assisted counting and ImageJ-assisted manual colony enumeration. Manual timing represented colony clicking time on digital images, whereas AI timing represented software-based image analysis time. The timing comparison did not include plate handling, technologist review, or manual correction, and therefore should not be interpreted as end-to-end workflow turnaround time.

AI-based counting was rapid and consistent, requiring approximately 5–15 s/plate for software-based image analysis. This processing time did not include plate handling, technologist review of overlays, manual correction, or result reporting. AI processing time showed no meaningful dependence on the number of colonies. By contrast, manual counting time increased with colony number in the recorded timing dataset. The relationship was defined by the formula Y = 0.68X + 6.89 (R^2^ = 0.94), indicating that manual counting time increased by approximately 0.67 s for each additional colony. Manual counting was faster than AI only for plates containing fewer than approximately 10 colonies; for plates with moderate or high colony burden, AI counting provided a substantial software analysis time advantage (Figure 5).

### 3.7. Residual Visual Challenges and Potential Clinical Failure Modes

**Figure 6:**
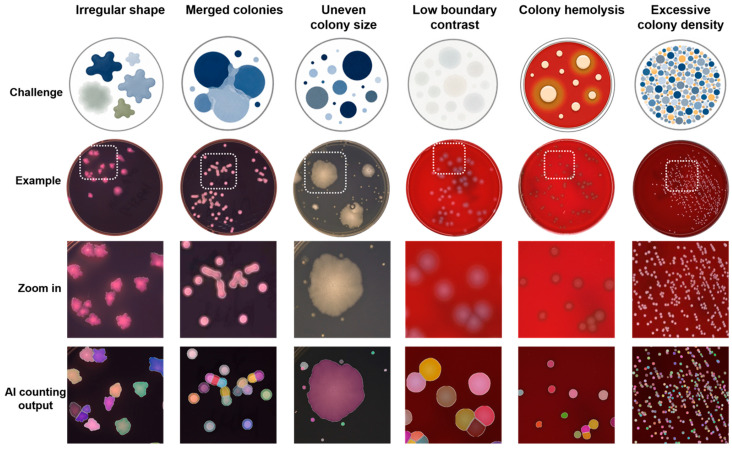
Representative visual challenges affecting colony counting. Representative examples of common colony counting challenges are shown. Irregular colony shape, including non-circular, lobated, or diffuse contours, can confuse shape-based detection and splitting. Merged colonies occur when adjacent colonies touch or coalesce, causing multiple colonies to be counted as one. Uneven colony size can obscure small colonies and complicate thresholding or size-based filtering. Low boundary contrast can lead to missed or poorly segmented colonies when colony edges are faint or similar to the background. Hemolytic halos on blood agar can alter local appearance and interfere with boundary recognition. Excessive colony density reduces separation between colonies and increases counting uncertainty across the field. White dashed boxes indicate the regions enlarged in the corresponding “Zoom in” panels below. Colors in the AI-counting output are arbitrary pseudo-colors assigned to distinguish individual segmented colony instances and do not represent microbial species, colony type, confidence score, or quantitative value.

Representative visual challenges are summarized in Figure 6. These included irregular colony shape, merged or adherent colonies, uneven colony size, low boundary contrast, hemolytic halos on blood agar, and excessive colony density.

A post hoc review was further performed for the 22 plates with an absolute AI–manual discrepancy greater than 10 CFU. The most frequent dominant visual factors were merged or adherent colonies, observed in 9/22 (40.90%) discrepant plates, and excessive colony density or very small colonies, observed in 9/22 plates (40.90%). Irregular colony morphology, uneven colony size, low boundary contrast, and hemolytic colonies each accounted for 1/22 discrepant plates (4.55%). Thus, colony separation- and density-related factors, including adherent colonies and excessive colony density or very small colonies, accounted for 18/22 discrepant plates (81.82%). These findings indicate that residual AI–manual disagreement was concentrated mainly in plates where colony separation was intrinsically difficult or where very dense or very small colonies increased detection uncertainty.

### 3.8. Additional Error-Based Agreement Analyses

To avoid overreliance on correlation-based measures, additional error-based and agreement analyses were performed for the 382 paired AI–manual median records (Table 2 and Table 3). The MAE was 3.19 CFU/plate (95% CI, 2.68 to 3.78), the median AE was 1.00 CFU/plate, and the RMSE was 6.57 CFU/plate (95% CI, 5.29 to 7.81). The median relative absolute error was 1.07%. The Lin concordance correlation coefficient was 0.99 (95% CI, 0.99 to 0.99), and the absolute agreement ICC was also 0.99 (95% CI, 0.99 to 0.99). Passing–Bablok regression yielded Manual count = 0.04 + 0.993 × AI count, with a slope 95% CI of 0.99 to 0.99. When the analysis was stratified by colony density, the absolute error increased with higher colony burden, but the median relative absolute error remained low across density strata (Table 2). Predefined four-category plate count agreement was observed in 372/382 paired records (97.38%); 10 records (2.63%) shifted by one adjacent category, and no record shifted by two or more categories. Cohen’s kappa was 0.963 (95% CI, 0.94 to 0.98), and the quadratic weighted kappa was 0.99 (95% CI, 0.98 to 0.99).

## 4. Discussion

The accurate enumeration of microbial colonies is a fundamental yet time-consuming and subjective task in clinical microbiology. This study demonstrates that, in standardized pure culture digital plate images, the AI-assisted colony counting workflow provided close agreement with ImageJ-assisted manual comparator counts across a wide spectrum of colony densities, morphologies, and culture media. Therefore, the findings should be interpreted as a method comparison evaluation against a designated manual comparator, rather than as proof of absolute counting truth or full clinical deployment readiness.

Colony counting remains a clinically relevant but operator-dependent procedure. In routine clinical microbiology, quantitative or semi-quantitative colony counts are used in settings such as urine culture, bronchoalveolar lavage culture, catheter-related culture, and other quantitative culture workflows, where the final interpretation may depend on specimen type, collection method, and reporting threshold [3,4,5,6]. However, manual counting is influenced by reader experience, local counting rules, colony aggregation, and fatigue, while automated plate-reading systems can still produce counting errors or require visual correction when colony separation, colony contrast, or plate background is unfavorable [1,9,15,16]. In this context, the present study provides a positive evaluation of a reviewable AI-assisted workflow, showing that large AI–manual discrepancies were uncommon and that the AI output could be visually inspected rather than accepted as an unreviewed number.

A pivotal finding is the Starry-300 AI system’s proficiency across an exceptionally broad range of colony densities, a known challenge for both manual and automated colony enumeration [1,9,19,27]. Previous research has indicated that intelligent counting can struggle with very low (<10 CFU) or very high (>300 CFU) colony counts, with substantial counting errors under these conditions [9]. In contrast, our data show that the regression slope remained close to 1.0 for both low-count (0–150 CFU) and high-count (>300 CFU) plates. This performance compares favorably with some earlier automated approaches, which often showed reduced accuracy when colonies became crowded or poorly separated [9,19,27]. The observed tendency for AI to slightly overcount, by an average of approximately one colony per plate, is consistent with some previous observations in automated counting, but the magnitude was markedly reduced, with only 1.31% of paired plates exceeding a 30 CFU difference [9].

The system’s ability to handle complex colony morphologies is another significant advancement. Colonies may be mucoid, adherent, transparent, hemolytic, small, irregular, or mixed in size, and culture media differ in color, opacity, surface reflectance, and background contrast. The AI system effectively segmented adherent colonies, such as those of mucoid *K. pneumoniae*, achieving correlation coefficients > 0.95. This is particularly notable because studies of clinical culture automation and AI-assisted urine culture analysis have shown that colony adjacency, weak separation, and visually ambiguous growth can reduce counting or interpretation accuracy [15,16,19,27]. The AI color coding of adhesion levels provides a quantitative visual cue for colony confluence that is difficult to ascertain manually. Furthermore, the dual-illumination strategy and multi-stage image analysis pipeline were designed to improve colony background contrast and to handle both small- and large-colony regions. The system demonstrated reliable detection of small-colony Gram-positive bacteria, indicating its ability to resolve microcolonies, a task where some large-scale automation systems have reported decreased identification rates [15,16].

The impact of different culture media on counting precision was minimal. The high correlation observed on NA, BAP, MAC and SDA suggests that the system’s use of dual-light sources effectively mitigates variations in agar opacity and color. Culture medium, incubation, and colony growth conditions have been reported to influence colony appearance and image clarity, which may affect both manual and automated interpretation [28,29]. In addition, common plate artifacts such as scratches and bubbles were consistently ignored, addressing a frequent cause of overcounting in traditional automated devices and image analysis workflows [8,9,30,31,32].

Perhaps one of the most compelling arguments for AI adoption is the combination of deterministic repeat analysis and reduced software counting time. Our study uncovered inter-technologist variability, with 26/382 plates (6.81%) showing manual counting discrepancies exceeding 30 CFU. This subjectivity for confluent or high-density growth poses a risk to standardized reporting, and published observations have also shown that manual culture reading is not perfectly reproducible under routine conditions [33]. In terms of efficiency, the AI counting time was consistently brief and independent of colony density. In contrast, manual counting time increased linearly with colony numbers, representing a scalable labor burden in high-throughput settings [13,14].

The clinical relevance of residual counting discrepancies requires careful interpretation. The ±10 CFU and >30 CFU thresholds were pragmatic image-level analytical thresholds rather than clinical decision thresholds. A difference that is analytically small in a high-count pure culture plate may be clinically relevant if it shifts a near-threshold specimen across a reporting category or decision boundary. Therefore, AI-assisted counts near clinically important thresholds, such as urine culture or quantitative BAL thresholds, should be reviewed with the original image and detection overlay rather than accepted as an unreviewed numerical output [3,4,5,6]. Future studies should deliberately include near-threshold primary clinical specimens and evaluate whether AI-assisted counting changes reporting categories, clinical interpretation, or review requirements.

The failure mode review showed that residual discrepancies were not randomly distributed across visual patterns. Among the 22 plates with >10 CFU AI–manual discrepancies, adherent colonies and excessive colony density or very small colonies were the two dominant visual factors, together accounting for 18/22 (81.82%) discrepant plates. This indicates that colony separation and reliable detection of crowded or very small colonies remain the main sources of residual counting disagreement. These patterns can lead to undercounting when adjacent colonies are grouped as one object, or to missed detections when colonies are very small or poorly separated. In contrast, irregular colony morphology, uneven colony size, low boundary contrast, and hemolytic background effects were less frequent in this discrepant case review. Because this review was limited to plates with >10 CFU AI–manual discrepancies, the frequencies should be interpreted as error case frequencies rather than overall prevalence estimates.

This study has several limitations. First, all plates were generated from standardized pure culture isolates rather than primary mixed clinical specimens. No mixed-culture plates, polymicrobial growth patterns, contaminants, or specimen-derived debris were included, so performance in primary clinical cultures remains unknown. Second, all experiments were conducted at a single center using one Starry-300 imaging platform and one acquisition protocol; the robustness of the system across laboratories, operators and incubation conditions remains to be determined. Third, the yeast dataset was relatively small: only 26 SDA plates were included, and the yeast subset was limited to *C. albicans*. Therefore, the findings for yeast colony counting should be interpreted as preliminary. Fourth, the timing analysis was limited to software-based image analysis time for AI-assisted counting and ImageJ-assisted colony clicking time for manual counting. It did not include plate loading, technologist review of AI overlays, manual correction and result reporting. Therefore, the reported 5–15 s/plate AI analysis time should not be interpreted as end-to-end workflow turnaround time.

Finally, AI should not be presented as solving all colony counting problems. Residual errors can still occur in plates with severe adhesion, weak colony boundaries, hemolysis, mucoid or spreading growth, mixed colony sizes, structured backgrounds, or artifacts that resemble biological growth. Future studies should deliberately enrich datasets for these challenging scenarios and should evaluate primary specimens, mixed cultures, serial imaging during incubation, and additional culture formats used in clinical, food, pharmaceutical, and environmental microbiology. For clinical microbiology, near-threshold urine cultures and quantitative respiratory specimens should be prioritized because small enumeration differences may influence interpretation. Future work should also assess how technologists use AI overlays in review and correction workflows and whether AI-assisted counting improves between laboratory reproducibility. Similar expansion will be needed for chromogenic and organism-specific applications where subtle color or morphology differences are part of the interpretation [17,18,20].

## 5. Conclusions

In this standardized pure culture digital plate dataset, the Starry-300 AI colony counting system provided rapid deterministic image analysis and close agreement with ImageJ-assisted manual comparator counts across bacterial and the limited yeast subset on BAP, MAC, NA, and SDA. Agreement with manual counting remained strong across density strata, including >300 CFU/plate, and the AI workflow reduced the software analysis time burden associated with high-density manual enumeration. By combining automated counting with reviewable detection overlays, the system offers a practical AI-assisted approach for quantitative microbiology workflows. However, the residual visual challenges and study design limitations emphasize that AI counting should be implemented as a reviewable decision support tool, with further validation using primary mixed clinical specimens, near-threshold samples, external sites, and clinically important visually complex specimens before broad deployment.

## Figures and Tables

**Table 1:** Linear regression analysis between AI-assisted and manual colony counting across density strata, organism groups, and culture media.

Category	Subgroup	Number of Agar Plates, *n*	Linear Regression Equation	Slope 95% CI	Intercept 95% CI	R^2^
colony count per plate	0–150	258	Y = 0.98X + 0.31	0.98 to 0.99	−0.09 to 0.71	1.00
colony count per plate	151–300	58	Y = 0.92X + 12.46	0.89 to 0.96	3.82 to 21.10	0.98
colony count per plate	>300	66	Y = 0.99X + 3.75	0.97 to 1.01	−5.25 to 12.75	0.99
species	Gram-negative bacilli	232	Y = 0.99X − 0.42	0.99 to 1.00	−1.56 to 0.71	1.00
species	Gram-positive cocci	124	Y = 0.99X + 0.61	0.99 to 1.00	−0.73 to 1.94	1.00
species	Yeast	26	Y = 1.00X − 1.53	0.99 to 1.00	−4.99 to 1.92	1.00
culture media	BAP	128	Y = 0.99X + 0.28	0.98 to 0.99	−0.71 to 1.26	1.00
culture media	MAC	81	Y = 0.99X − 0.79	0.97 to 1.00	−3.47 to 1.88	0.99
culture media	NA	147	Y = 0.99X + 0.15	0.99 to 1.00	−1.01 to 1.31	1.00
culture media	SDA	26	Y = 1.00X − 1.53	0.99 to 1.00	−4.99 to 1.92	1.00
overall	Overall paired AI–manual records	382	Y = 0.99X − 0.26	0.99 to 1.00	−1.07 to 0.55	1.00

Note: AI–manual agreement analyses used the 382 plates with paired AI and primary manual comparator counts.

**Table 2:** Error metrics stratified by manual colony density category.

Manual Median Count Category	Mean Bias (AI − Median) (95% CI)	MAE (95% CI)	Median AE	RMSE (95% CI)	Median Relative Absolute Error (%)	>30 CFU Difference
0–150 CFU	+0.44 (0.15 to 0.73)	1.26 (1.02 to 1.52)	0.00	2.39 (1.93 to 2.84)	0.00%	0/258 (0.00%)
151–300 CFU	+4.24 (2.27 to 6.21)	5.34 (3.86 to 7.28)	3.50	8.57 (5.31 to 11.82)	1.62%	1/58 (1.72%)
>300 CFU	+2.06 (−1.06 to 5.18)	8.85 (6.74 to 11.44)	7.00	12.78 (9.60 to 15.72)	1.26%	4/66 (6.06%)
Total	+1.30 (0.65 to 1.95)	3.19 (2.68 to 3.78)	1.00	6.57 (5.29 to 7.81)	1.07%	5/382 (1.31%)

Note. MAE, mean absolute error. Median AE, median absolute error. RMSE, root mean square error.

**Table 3:** Additional agreement statistics between AI-assisted counts and the three-reader median manual comparator.

Metric	Value	95% CI/Description
ICC, absolute agreement	0.99	0.99 to 0.99
Lin’s concordance correlation coefficient	0.99	0.99 to 0.99
Passing–Bablok regression	Manual = 0.04 + 0.993 × AI	0.99 to 0.99
Cohen’s kappa	0.96	0.94 to 0.98
Quadratic weighted Cohen’s kappa	0.99	0.98 to 0.99

## Data Availability

The raw data supporting the conclusions of this article will be made available by the authors on request.

## Supplementary Materials

The following supporting information can be downloaded at: https://www.mdpi.com/article/10.3390/microorganisms14071426/s1, Table S1. Architecture, training settings, and dataset sizes of the eight submodels in the Starry-300 AI colony-counting pipeline; Table S2. Plate-count category agreement between AI-assisted and manual colony counts.
