# Supplementary material for: Evaluation of an AI-Assisted Colony Counting System Across Multiple Culture Media Using Standardized Pure Culture Plates
Source: Microorganisms. 2026 Jun 30;14(7):1426. doi: 10.3390/microorganisms14071426 (PMC13413662; doi:10.3390/microorganisms14071426)
Supplement: Supplementary file 1 [file microorganisms-14-01426-s001.zip › microorganisms-4362367-supplementary.pdf]

Evaluation of an AI-Assisted Colony Counting System Across Multiple Culture Media Using Standardized Pure Culture Plates

Supplementary Table S1. Architecture, training settings, and dataset sizes of the eight submodels in the Starry-300 AI colony-counting pipeline

Submodel	Pipeline role / task	Model configuration				Training settings			Dataset size	
		Architecture	Backbone / pretraining	Input resolution	Input channels	Batch	Epochs	Params (M)	Train（n)	Valid (n)
**disk_segs**	Petri-dish disk segmentation for ROI preprocessing	U-Net	timm-regnetx_008, ImageNet pretrained	512 x 512	3, top reflected-light RGB only	8	100	10.21	1,165	292
**all_segs**	Whole-plate semantic segmentation	U-Net++	timm-regnetx_008, ImageNet pretrained	1024 x 1024	6, top reflected + bottom transmitted RGB	4	50	11.75	4,655	1,165
**small_segs**	Small-colony semantic segmentation	U-Net	timm-regnetx_008, ImageNet pretrained	512 x 512	6, top reflected + bottom transmitted RGB	8	50	10.17	37,940	8,998
**edge**	Boundary-refinement model for colony edges	U-Net	timm-regnetx_008, ImageNet pretrained	1024 x 1024	6, top reflected + bottom transmitted RGB	4	50	10.17	12,001	3,001
**large_insts**	Large-colony instance segmentation	YOLOv8n-seg, 6-channel	CSPDarknet (C2f + SPPF, scale n), COCO pretrained	512 x 512	6, top reflected + bottom transmitted RGB	4	100	3.26	10,438	2,600
**small_insts**	Small-colony instance segmentation	YOLOv8n-seg, 6-channel	CSPDarknet (C2f + SPPF, scale n), COCO pretrained	128 x 128	6, top reflected + bottom transmitted RGB	16	100	3.26	24,430	6,108
**stage1_5cls**	First-stage coarse colony morphology classification, 5 classes	YOLOv8s-cls	CSPDarknet (scale s), ImageNet pretrained	1024 x 1024	3, top reflected-light RGB only	16	100	5.09	13,144	3,286
**stage2_8cls**	Second-stage fine colony morphology classification, 8 classes	YOLOv8s-cls	CSPDarknet (scale s), ImageNet pretrained	1024 x 1024	3, top reflected-light RGB only	16	100	5.09	13,270	3,317

Note. ROI, region of interest; RGB, red-green-blue; SPPF, spatial pyramid pooling fast; COCO, Common Objects in Context. Params are shown in millions. Three-channel inputs use only the top reflected-light RGB image, whereas six channel inputs concatenate top reflected light and bottom transmitted light RGB images. Training and validation sample counts refer to model-specific image patches, crops, masks, or classification samples used for the corresponding subtask.

Supplementary Table S2. Plate-count category agreement between AI-assisted and manual colony counts.

Manual category	AI 0–50 CFU	AI 51–150 CFU	AI 151–300 CFU	AI >300 CFU	Row total (n)
Manual 0–50 CFU	158	3	0	0	161
Manual 51–150 CFU	1	95	1	0	97
Manual 151–300 CFU	0	0	55	3	58
Manual >300 CFU	0	0	2	64	66
Column total (n)	159	98	58	67	382
